# Neural circuits: Japan

**DOI:** 10.3389/fncir.2014.00135

**Published:** 2014-11-10

**Authors:** Yasuo Kawaguchi, Masanobu Kano

**Affiliations:** ^1^Division of Cerebral Circuitry, National Institute for Physiological SciencesOkazaki, Japan; ^2^Department of Neurophysiology, Graduate School of Medicine, The University of TokyoTokyo, Japan

**Keywords:** neocortex, hippocampus, olfactory bulb, basal ganglia, hypothalamus, cerebellum, Drosophila, *C. elegans*

This Frontiers Research Topic on “Neural Circuits: Japan” explores the diversity of innovative neural circuit research occurring across Japan. This issue brings together papers revealing the development, structure, and physiology of neuronal circuits involved in sensory perception, sleep and wakefulness, behavioral selection, and motor command generation in a range of species, from nematode to primate.

One area of interest includes cerebellar local and efferent circuits, as well as mechanisms underlying synaptic plasticity in Purkinje cells, which have been a focus of intensive investigation by many Japanese researchers. Long-term depression at connections between parallel fibers and Purkinje cells is thought to play a critical role in motor learning. In mice without Cbln1, delay eye blink conditioning, as well as LTD at the parallel fiber to Purkinje cell synapse, were impaired, even as previously formed conditioning responses were retained, suggesting a necessity for LTD selectively in conditioning formation (Emi et al., 2013). The cerebellar cortex is composed of functionally different microzones. Investigation of microzonal organization in glutamate receptor δ2 knockout mice revealed that proper innervation by individual climbing fibers is necessary for functional microzone formation (Hashizume et al., 2013). In dystonia, the cerebellum as well as the basal ganglia may be involved in its pathogenesis. Deletion of type 1 inositol 1,4,5-trisphosphate receptors in the cerebellum induced dystonic movements and abnormal movement-coupled firing in Purkinje cells, while inactivation of the inferior olive suppressed abnormal movements. These results suggest the involvement of the olivo-cerebellar pathway in dystonia (Hisatsune et al., 2013).

For analysis of synaptic plasticity in the cerebral cortex, diverse imaging techniques and optical probes have been used. Among them, Förster resonance energy transfer (FRET), combined with newly developed probes, will greatly contribute to our understanding of synaptic plasticity mechanisms at the molecular level (Ueda et al., 2013). During normal formation of cortical circuits, axons and dendrites need to generate boutons and spines at proper locations, respectively. In the cerebral cortex, collateralization of thalamocortical fibers depends on concentrations of brain-derived neurotrophic factor (BDNF), and this BDNF-dependent collateralization was absent after suppression of synaptic vesicle recycling (Granseth et al., 2013). In hippocampal CA3 neurons, application of corticosterone increased the density of dendritic thorny excrescences in pyramidal cells for short time. Similarly, acute stress increases the connectional strength of dentate gyrus to CA3 synapses (Yoshiya et al., 2013). Neocortical pyramidal cells are composed of multiple subtypes that differ in their subcortical projection targets. The subtype composition of layer 5 pyramidal cells connecting cortical areas is also variable, and depends on the target area (Ueta et al., 2013). These observations suggest that cortical neurons are selectively connected according to their individual identities. Further, the temporal pattern of spike discharges may reflect the connectional selectivity of cortical neurons. Large scale activity recordings in the CA3 region of the hippocampus have revealed that temporal firing sequences among a given group of pyramidal cells are repeated among spontaneous spikes of hippocampal neurons *ex vivo* (Matsumoto et al., 2013).

Compared to projections from the retina to primary visual cortex (V1), connections between higher visual centers are less understood. In mice, axons from V1 respond differently to visual input depending on their projection to higher visual areas (Matsui and Ohki, 2013). These results suggest that different V1 cells with distinct visual responses project to different higher visual areas. In *Drosophila*, analysis of visual stimuli-dependent behaviors revealed visual response differences among higher visual centers (Otsuna et al., 2014). On the other hand, in the olfactory system, newly generated neurons are continually integrated into neuronal circuits, and the survival of new neurons is dependent on sleep following food consumption. The olfactory bulb and cortex are suitable for the analysis of sleep-dependent plastic mechanisms in the brain (Yamaguchi et al., 2013).

To understand the switching mechanism between sleep and wakefulness, it is necessary to understand the synaptic interactions between hypothalamic nuclei participating this process. Optogenetic analysis has revealed inhibitory connections from GABAergic cells in the preoptic area to Orexin cells in the lateral hypothalamic area (Saito et al., 2013). The planning and execution of behavior require computation in the basal ganglia and frontal cortex, as well as cortical output to the spinal cord. Conventional electrophysiological and anatomical methods have proven insufficient to clarify these complex circuits. On the other hand, the combined use of local transfection and retrograde transport of viruses were able to block synaptic outputs in the crossed tecto-reticular pathway to suppress motor function (Sooksawate et al., 2013). The combination of retrogradely-transported virus and immunotoxins have also successfully blocked synaptic transmission from motor-related cortical areas to the subthalamic nucleus (Takada et al., 2013). Pathway-selective inhibition using retrogradely-transported viruses will continue to be a critical tool for elucidating the functions of individual projection systems. The connection loops formed by the basal ganglia and frontal cortex participate in selection of proper movements depending on sensory information. The globus pallidus and frontal areas, such as the dorsal premotor cortex, the dorsolateral prefrontal cortex, and the ventrolateral prefrontal cortex, participate in setting behavioral goals according to cues in the external environment (Hoshi, 2013).

On the other hand, *C. elegans* is an excellent model organism for circuit function analysis because its neuronal organization has been well-characterized, and diverse genetic manipulations are easily achievable. Using *C. elegans*, Japanese researchers have made significant contributions to the understanding of neural circuit that generate behaviors in response to sensory information such as odor and temperature (Sasakura et al., 2013). Thus, as in the USA and Europe, researchers in Japan are now focusing efforts to elucidate the function of neural circuits in diverse organisms by taking advantage of optogenetics, genetic manipulations, and traditional physiological and anatomical approaches, as well as neural pathway-selective inactivation techniques that have recently been developed in Japan.

## Conflict of interest statement

The authors declare that the research was conducted in the absence of any commercial or financial relationships that could be construed as a potential conflict of interest.
